# Real-time PCR of CD146 mRNA in peripheral blood enables the relative quantification of circulating endothelial cells and is an indicator of angiogenesis

**DOI:** 10.1038/sj.bjc.6602782

**Published:** 2005-09-06

**Authors:** G Fürstenberger, R von Moos, H-J Senn, E-M Boneberg

**Affiliations:** 1 Center for Tumor Detection and Prevention, Rorschacherstrasse 150, St Gallen 9006, Switzerland; 2 Senology Center of Eastern Switzerland, St. Gallen, St Gallen 9006, Switzerland; 3 Biotechnology Institute Thurgau, Taegerwilen 8274, Switzerland

**Keywords:** real-time PCR, angiogenesis, circulating endothelial cells, CD146, breast cancer

## Abstract

Angiogenesis is a fundamental process in tumour growth and metastatic dissemination. Possible surrogate markers for tumour angiogenesis are the amounts of circulating endothelial cells (CEC) in peripheral blood and the plasma concentration of vascular endothelial growth factor (VEGF). We tested the suitability of real-time PCR for CD146, an endothelial cell-specific antigen, to quantify CEC numbers in comparison to a flow cytometry quantification. Real-time PCR of CD146 mRNA showed high sensitivity and linearity for the quantification of cultivated primary endothelial cells added in different amounts to blood samples. Circulating endothelial cell numbers were quantified in peripheral blood samples of breast cancer patients and healthy controls by four-colour flow cytometry analysis and CD146 real-time PCR, and VEGF plasma concentrations were measured by ELISA. The amounts of CEC detected with both methods correlated significantly and CEC numbers were significantly increased in newly diagnosed breast cancer patients compared to healthy controls. Vascular endothelial growth factor concentrations correlated significantly with CEC numbers, but there was no significant difference in VEGF levels between breast cancer patients and healthy controls indicating that VEGF plasma levels cannot be used as surrogate marker for tumour angiogenesis. Taken together, the quantification of CEC by CD146 real-time PCR showed equivalent results to the flow cytometry analysis. Thus, CD146 real-time PCR may be an easy and reliable approach to quantify CEC in peripheral blood samples and could facilitate the integration of CEC measurements in clinical studies exploring the efficacy of antiangiogenic therapies.

Angiogenesis is a key step in tumour progression including the spread and growth of metastases. Most tumours start growing as avascular nodules until they reach a steady-state level of proliferating and apoptosing cells. The tumour stays in this dormant phase until angiogenesis is initiated and enables further tumour growth. This angiogenic switch starts with the detachment of pericytes and vessel dilation. The degradation of the basement membrane and extracellular matrix allows endothelial cells to migrate into the perivascular space towards chemotactic angiogenic stimuli. The endothelial cells then multiply, and finally differentiate and adhere to each other to form a lumen, which is accompanied by basement membrane formation and pericyte attachment (Ruoslahti, 2002; Bergers and Benjamin, 2003).

Tumours may also use alternative ways to obtain blood supply: Vessel co-option, the use of pre-existing vessels, was first described in the brain, but could also take place in other tumours (Leenders *et al*, 2002).

Endothelial cells may appear in the circulation by detaching from activated or damaged vessels. An increase of circulating endothelial cells (CEC) is described in several pathologic conditions that involve vascular injury or instability as myocardial infarction, infectious vasculitis and cancer (Mutin *et al*, 1999; Mancuso *et al*, 2001; Woywodt *et al*, 2003; Beerepoot *et al*, 2004). These CEC are mostly viable and exhibit still proliferative capacity despite their terminal differentiation (Lin *et al*, 2000; Mancuso *et al*, 2001; Ribatti, 2004).

Antiangiogenic therapy is a promising new form of cancer treatment and the effectiveness of new angiogenesis inhibitors are currently tested in many clinical studies. Surrogate markers for angiogenesis would be useful tools to study the effectiveness of antiangiogenesis drugs. Since it is suggested that endothelial cells are detached from the activated vessel wall during the formation of new vessels the number of CEC in peripheral blood could reflect the amount of proceeding neoangiogenesis and thus could serve as a surrogate marker for angiogenesis. Therefore methods to quantify CEC are of great importance. Quantification of CEC by flow cytometry is technically complex and other reliable and easy methods to quantify CEC should be developed. One promising approach is the application of real-time PCR to quantify endothelial cell-specific mRNA in blood samples. A possible candidate is mRNA coding for VE-cadherin as shown by Rabascio *et al* (2004). Another possible candidate for such a real-time approach is the mRNA coding for CD146.

CD146 (also referred to as MUC18, MCAM, Mel-CAM, S-Endo-1, P1H12 antigen) was initially identified as a marker of tumour progression and metastasis formation in human melanoma (Lehmann *et al*, 1989; Bardin *et al*, 1996; Shih *et al*, 1997). CD146 is homologous to several cell adhesion molecules and belongs to the immunoglobulin superfamily containing five extracellular immunoglobulin-like domains, a single transmembrane domain and a short cytoplasmic domain (Sers *et al*, 1993). Beside its expression in malignant melanocytes, CD146 is constitutively expressed in all endothelial cells, irrespective of the anatomical localisation (Bardin *et al*, 1996).

Several factors are involved in the control of angiogenesis. The most potent and specific is vascular endothelial growth factor (VEGF) (reviewed in Ferrara, 2000). Vascular endothelial growth factor seems to play a major role in tumour neoangiogenesis: transfection of tumour cells with VEGF results in enhanced angiogenesis and tumour growth in animal models (Zhang *et al*, 1995), antibodies blocking VEGF bioactivity inhibit tumour growth (Kim *et al*, 1993) and VEGF expression correlates with the degree of tumour vascularisation and the prognosis of several cancers (Vlaykova *et al*, 1999; Han *et al*, 2001). Therefore, VEGF measurements in peripheral blood are often performed as surrogate markers for angiogenesis (Tamura *et al*, 2001; Shimanuki *et al*, 2005).

In this study, we searched for a reliable surrogate marker for tumour angiogenesis: We tested the sensitivity of flow cytometry and real-time PCR to quantify endothelial cells in blood samples and compared the amounts of CEC in peripheral blood of breast cancer patients and healthy volunteers detected with both methods. Further we analysed VEGF plasma levels in these samples and correlated these values with the CEC numbers.

## MATERIALS AND METHODS

### Cell culture

Human microvascular lung endothelial cells (HMVEC-L) were purchased from Clonetics (Cambrex Bio Science, Walkersville, MD, USA) and maintained in 75 cm^2^ flasks (Integra Biosciences, Fernwald, Germany) with endothelial basal medium (EBM-2, Clonetics) supplemented with EGM-2 SingleQuots (Clonetics) containing fetal bovine serum, human VEGF, human FGF-B, human EGF, human R3-IGF-1, ascorbic acid, heparin, hydrocortisone and gentamycin/amphotericin-B. Cells were subcultured when they reached 70–90% confluence and were splitted 1 : 2, usually twice a week. Cells were only used until passage 15 as recommended by Clonetics.

### Patients and sample collection

This study was approved by the regional ethic board of St Gallen, Switzerland, and all patients and controls gave written informed consent before study entry. For the comparison of CEC numbers measured by flow cytometry or real-time PCR 50 blood samples (25 samples from healthy females, five samples from healthy males, 18 samples from female breast cancer patients (newly diagnosed or under treatment), two female patients with fibroadenoma) were analysed. For the comparison of breast cancer patients and healthy controls the 10 newly diagnosed female breast cancer patients and 11 healthy females with similar age were selected from the 50 samples analysed in this study. These patients were classified to the following pTNM staging groups: one patient stage 0, four patients stage I, two patients stage II and three patients stage III. Peripheral blood was collected with EDTA vacutainers (BD Biosciences, Allschwil, Switzerland).

### Flow cytometry

For measurement of CEC, a method from Mancuso *et al* (2001) was adapted. Mature CEC were defined as negative for haematopoietic marker CD45 and positive for endothelial markers CD146, CD31 and CD34. For the flow cytometric analysis 100 *μ*l EDTA blood was stained with 5 *μ*l anti-CD31-FITC (clone WM59, Serotec, Oxford, UK), 1 *μ*l P1H12-PE (clone P1H12, Chemicon, Dietikon, Switzerland), 5 *μ*l anti-CD45-PerCP (clone 2D1, BD Biosciences, Allschwil, Switzerland) and 5 *μ*l anti-CD34-APC (clone 8G12, BD Biosciences). For quantification of the CEC a known amount of 6 *μ*m latex microspheres (Polyscienes, Eppelheim, Germany) was added to the blood as an internal standard. After incubation for 30 min at room temperature, 1 ml BD Lysing Solution (BD Biosciences) was added to lyse erythrocytes and fixate cells. After 10 min, cells were washed twice with 1 ml PBS and measured in a FACS LSR flow cytometer (BD Biosciences) using Cell Quest software (BD Biosciences). In each blood sample 300 000 events were counted.

To test the sensitivity and linearity of flow cytometric detection of CEC, HMVEC-L were detached from the cell culture flask with 0.025% Trypsin/0.01% EDTA for 1 min. After washing in PBS/10% FCS cells were stained with P1H12-PE for 30 min at room temperature. Then the cells were washed twice with PBS, counted and added in different amounts to the EDTA blood samples. To the blood samples the latex microspheres were added and then blood was lysed and washed as described above. Since the cultured endothelial cells were stained before the addition to whole blood, staining with P1H12-PE was sufficient to identify these cells, whereas for the identification of CEC a complex four colour is necessary, since some cell populations in blood are positive for single endothelial markers.

### RNA isolation

In all, 0.5 ml EDTA blood was mixed with 14 ml of erythrocyte lysis buffer (0.899% (w/v) ammonium chloride, 0.1% (w/v) potassium bicarbonate, and 0.0037% (w/v) EDTA, pH 7.3) and incubated for 10 min at room temperature. After 10 min centrifugation with 500 **g** the buffer was removed and the cell pellet was resuspended in 350 *μ*l RLT buffer (Qiagen, Basel, Switzerland) and stored at −20°C until RNA isolation. RNA was isolated with the RNeasy Kit plus additional Dnase digestion (Qiagen) according to the manufacturer's instructions. RNA was eluted in 50 *μ*l RNase-free water. The quality of the isolated RNA was checked by gel electrophoresis.

### Reverse transcription

Samples for real-time PCR were reverse transcribed with the Taqman Reverse Transcription Kit from Applied Biosystems, Rotkreuz, Switzerland, according to the manufacturer's protocol. Samples were reverse transcribed with random hexamer primers and the maximal allowed volume of RNA sample that can be added to the reverse transcription reaction was used. To verify that the primers do not amplify genomic DNA, some RNA samples were diluted and incubated as the normal samples, but did not receive reverse transcriptase (RT samples).

### Primers

PCR primers for CD146 (NCBI reference sequence M28882) were designed with Primer Express software (Applied Biosystems) and were placed in two different exons (exons 2 and 6) to eliminate amplification of genomic DNA. The following primers for CD146 were used: forward primer 5′-cca agg caa cct cag cca tgt c-3′ and reverse primer 5′-ctc gac tcc aca gtc tgg gac gac t-3′. The resulting amplicon had a size of 437 bp.

### Real-time polymerase chain reaction

For the amplification of CD146 1 *μ*l cDNA was added to QuantiTect SYBR Green PCR Master Mix (Qiagen) containing 400 nM forward as well as reverse primers. PCR was performed in a Taqman 7700 (Applied Biosystems) using the following thermal settings: one cycle of 15 min at 95°C, and 40 cycles of 15 s at 94°C, 30 s at 60°C and 30 s at 72°C.

Relative mRNA expression was calculated with the ΔΔ*C*_t_-method. As calibrator the sample with the lowest *C*_t_-value was used and set to 100%.

To test the sensitivity and linearity of PCR detection of CEC, HMVEC-L were detached from the cell culture flask with 0.025% Trypsin/0.01% EDTA for 1 min. After washing in PBS/10% FCS cells were counted and added in different amounts to 0.5 ml EDTA blood and RNA was isolated as described above.

### ELISA measurement

Plasma was prepared from EDTA blood samples by centrifugation for 10 min at 14000 **g**. Plasma levels of VEGF were quantified by sandwich ELISA using the DuoSet ELISA Kit from R&D Systems (Wiesbaden, Germany) according to the manufacturer's instructions. Vascular endothelial growth factor measurements were performed in plasma and not in serum samples, since VEGF is released from activated platelets during the coagulation process (Hormbrey *et al*, 2002).

### Statistical analysis

Statistical analysis was performed using the GraphPad Instat software (Instant Statistics, GraphPad Software, San Diego, USA). For all data, unpaired nonparametric testing was performed with the Mann–Whitney test. Correlation testing was carried out with the Pearson's correlation analysis.

## RESULTS

### Sensitivity and linearity of CEC detection by real-time PCR

**Figure 1:**
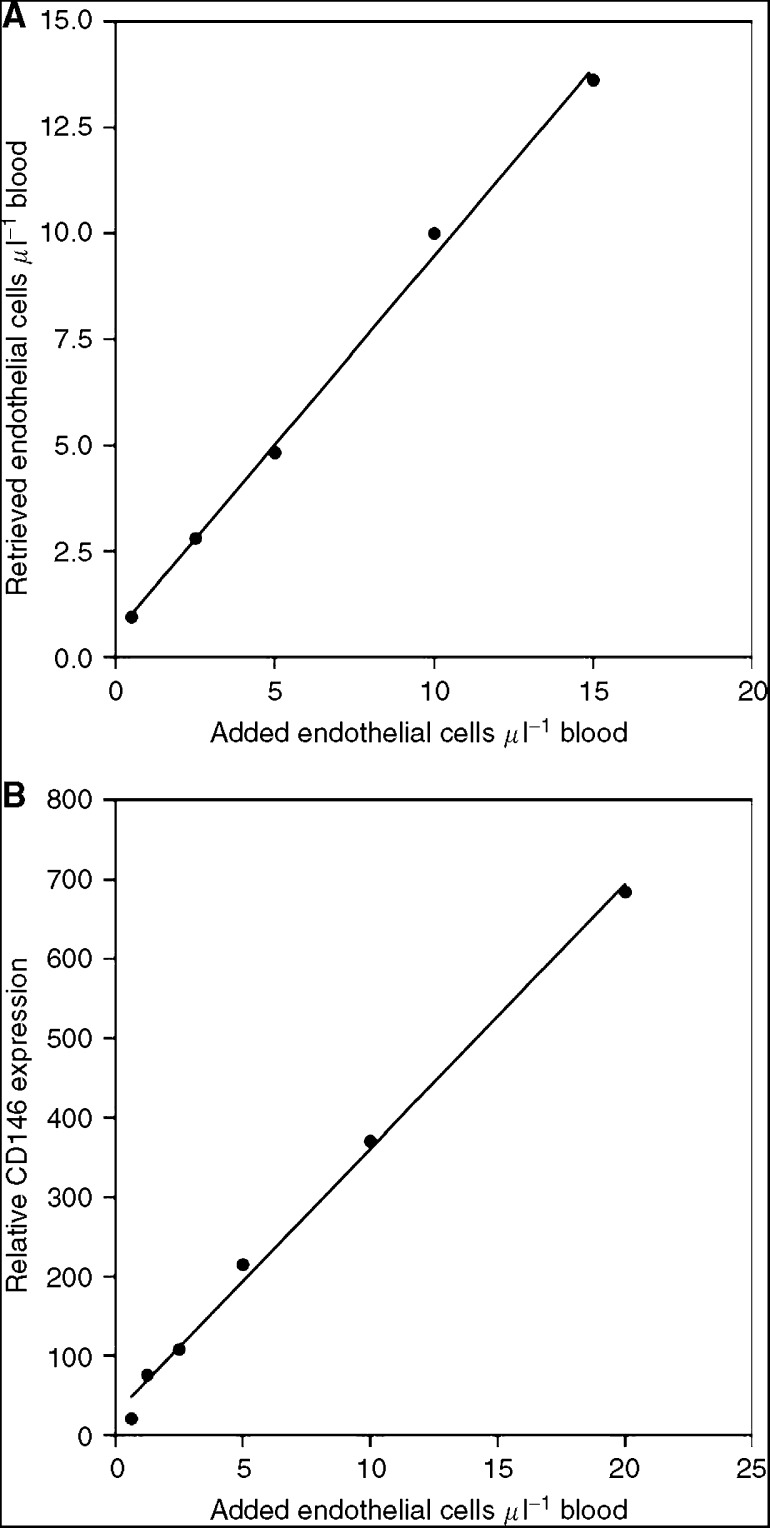
Sensitivity and linearity of the detection of endothelial cells in blood samples by flow cytometry and CD146 real-time PCR. Cultured human endothelial cells (HMVEC-L) were added in different amounts to blood and then the amounts of endothelial cells were determined with (**A**) four-colour flow cytometry analysis and (**B**) CD146 real-time PCR.

The standard method to detect CEC is multicolour flow cytometry. To compare the flow cytometric detection of endothelial cells with real-time PCR, we added different amounts of cultured human endothelial cells (HMVEC-L) to blood and analysed this by flow cytometry and real-time PCR for CD146. With the flow cytometric approach 0.5 added endothelial cells *μ*l^−1^ blood could be detected and the amounts of added endothelial cells correlated highly (*r*=0.9981, *P*<0.001) with the amounts of endothelial cells retrieved by flow cytometric analysis (Figure 1A).

For the quantification of endothelial cells by real-time PCR, we measured the amounts of CD146 mRNA. This PCR approach showed a very high sensitivity, even the lowest amount of 0.6 HMVEC-L *μ*l^−1^ blood (*C*_t_-value: 31.5) was clearly different from the control blood sample without added HMVEC-L (*C*_t_-value: 35.3). The relative expression of CD146 in real-time PCR correlated highly (*r*=0.996, *P*<0.001) with the added amounts of HMVEC-L (Figure 1B). This comparison showed that the quantification of CD146 mRNA could be used to quantify endothelial cells in blood samples, even in the range of very low CEC amounts. The sensitivity and linearity of the PCR method was comparable to the flow cytometric analysis.

### Correlation of CEC amounts detected by flow cytometry and real-time PCR

**Figure 2:**
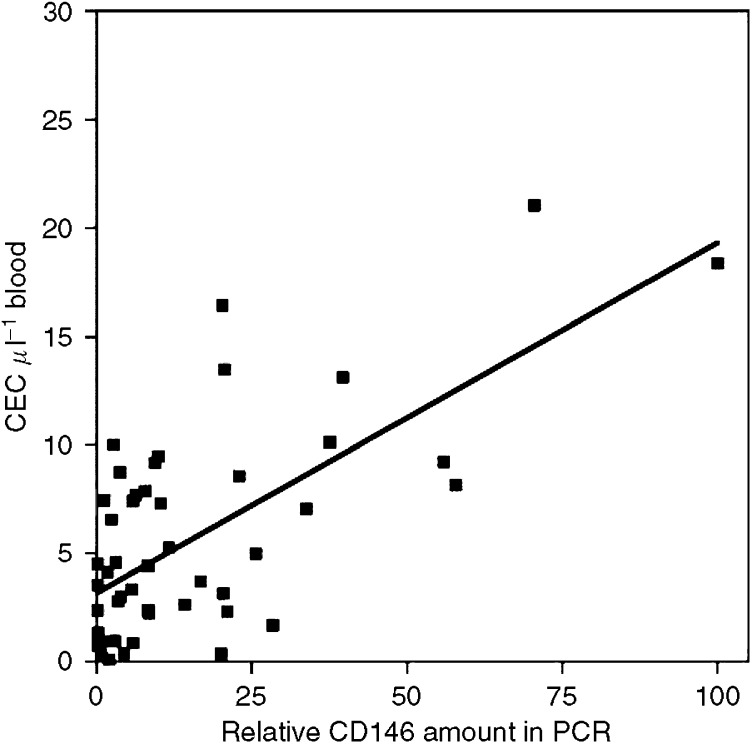
Correlation of CEC amounts detected by flow cytometry and CD146 real-time PCR. The amounts of CEC were quantified in 50 peripheral blood samples of patients and healthy controls by four-colour flow cytometry analysis and CD146 real-time PCR.

In blood samples of healthy controls and patients CEC were quantified in parallel by flow cytometry and CD146 real-time PCR. The amounts of CEC detected in 50 blood samples with both methods correlated significantly (*r*=0.6744, *P*<0.001) (Figure 2).

### Correlation of CEC amounts with plasma levels of VEGF

**Figure 3:**
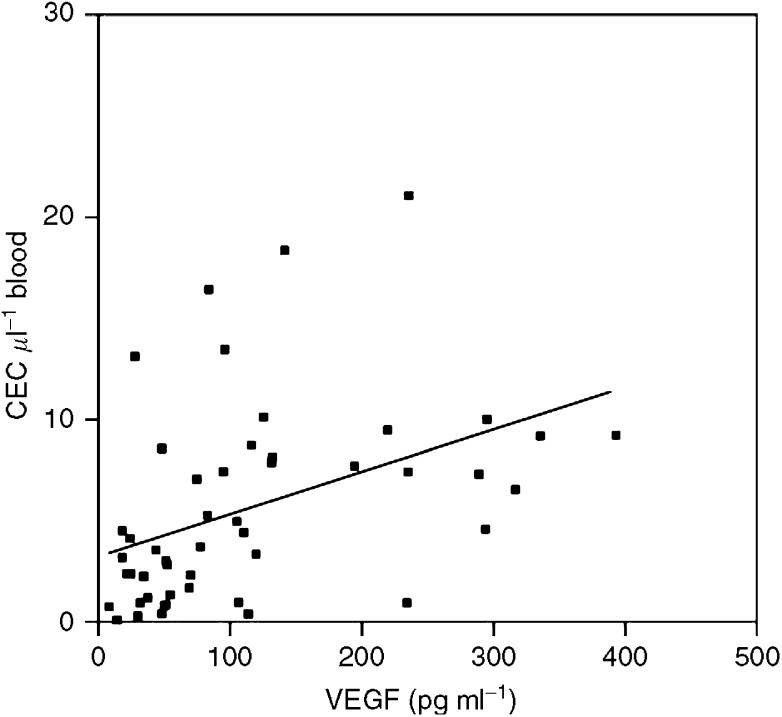
Correlation of CEC amounts and VEGF plasma levels. The concentrations of VEGF were quantified in 50 plasma samples of patients and healthy controls by ELISA and correlated with the amounts of CEC detected in these blood samples by four-colour flow cytometry analysis.

Since VEGF is one of the major cytokines triggering angiogenesis we also measured plasma levels of this cytokine in the blood samples of healthy controls and patients and correlated the plasma concentrations with the amounts of CEC detected by flow cytometry. Plasma levels of VEGF correlated significantly (*r*=0.4222, *P*=0.0025) with the amounts of CEC detected by flow cytometry (Figure 3).

### Comparison of CEC amounts and VEGF plasma levels in breast cancer patients and healthy controls

**Figure 4:**
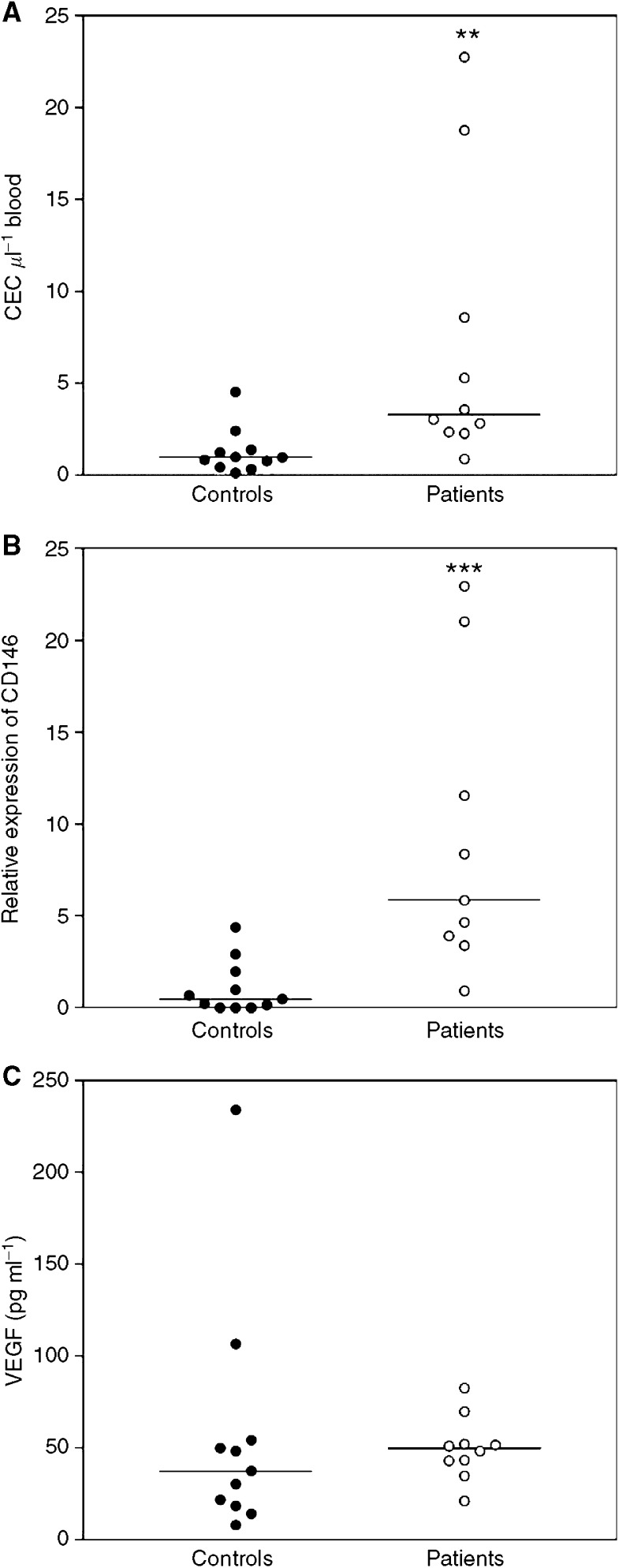
Comparison of CEC amounts and VEGF plasma levels in breast cancer patients and healthy controls. The amounts of CEC in peripheral blood samples of 11 healthy controls and 10 newly diagnosed breast cancer patients were determined by (**A**) four-colour flow cytometry analysis and (**B**) CD146 real-time PCR. (**C**) Concentrations of VEGF in the plasma samples of these patients and controls were quantified by ELISA ^**^P<0.01, ^***^P<0.001 *vs* values of healthy controls.

The amounts of CEC in peripheral blood of breast cancer patients and healthy controls were quantified by flow cytometry and real-time PCR of CD146. With flow cytometry 1.0 CEC *μ*l^−1^ blood (median) were detected in the blood samples of the healthy controls, whereas in the breast cancer patients at the time of diagnosis 3.3 CEC *μ*l^−1^ blood (median) were found (Figure 4A). This difference between patients and controls was significant (*P*=0.0021).

With real-time PCR the relative amounts of CEC were determined by quantification of CD146 mRNA in the blood samples. The relative amount of CEC in the control population was 0.47 (median), whereas the breast cancer patients had a relative CEC amount of 5.9 (median) (Figure 4B). This difference was also significant (*P*=0.0004).

VEGF was measured by sandwich ELISA in plasma of breast cancer patients and healthy controls. In healthy controls VEGF plasma levels were 37.4 pg ml^−1^ (median), in the breast cancer patients concentrations of 49.6 pg ml^−1^ (median) were reached (Figure 4C). This difference between healthy controls and patients was not significant.

## DISCUSSION

VEGF was originally identified for its ability to induce vascular permeability and to stimulate endothelial cell growth. Now it is recognised as a key factor required for tumour neoangiogenesis (Ferrara and Gerber, 2001). Currently, several inhibitors of the VEGF-signaling pathway have entered clinical trials to test their efficacy as antiangiogenic drugs (reviewed in Verheul and Pinedo, 2003). Unfortunately, clinical markers to assess neoangiogenesis in patients or the response to antiangiogenic treatment are still scanty. Circulating endothelial cell numbers in peripheral blood seem to be a relevant marker of neoangiogenesis since CEC are elevated in cancer patients and the amounts of CEC correlates with progressive disease (Mancuso *et al*, 2001; Beerepoot *et al*, 2004). The amounts of CEC in peripheral blood can be measured by flow cytometry, but this requires fresh blood samples and a complex four-colour analysis with the acquisition of many events. Another limitation of this method is the danger to falsely identify platelet aggregates as endothelial cells. We observed in some patient samples (three in 115) high numbers of events that were highly positive for the endothelial cell marker CD146, but a closer analysis of these events by staining the blood with the DNA dye Hoechst 33342 and flow cytometric analysis with UV laser showed that these events did not contain DNA and thus could not be endothelial cells. Since CD146 is found in a soluble form in human plasma (Bardin *et al*, 2003) these CD146-positive events could result from platelet aggregates which have bound soluble CD146 resulting in specific staining of these complexes with the anti-CD146 antibody.

Real-time PCR offers several advantages compared to flow cytometry. It is possible to freeze the blood samples facilitating the integration of such measurement in clinical routine work and the standardisation of the real-time PCR method is easier than a standardisation of the complex four-colour flow cytometry analysis. Further in flow cytometry there is always the danger of unspecific staining with antibodies, especially in patients with activated leucocytes, and the detection of platelet aggregates as cells. This could in particular influence the detection of such tiny populations as the CEC.

To overcome these limitations we searched for another method to quantify CEC in peripheral blood. We therefore tested real-time PCR for the endothelial cell marker CD146 to quantify CEC in blood samples. Since the flow cytometric measurement of CD146 showed a stable expression of CD146 on CEC, we assumed that the quantification of CD146 mRNA would allow to determine the amounts of CEC present in the blood samples. We therefore compared the sensitivity and linearity of flow cytometry and real-time PCR detection of endothelial cells. Cultivated primary endothelial cells added to blood samples were detected with both methods with a similar sensitivity and a strong linearity with increasing numbers of endothelial cells. Even low amounts of endothelial cells (below 1 cell *μ*l^−1^ blood) could be detected with both methods.

Next, we compared the amounts of CEC in peripheral blood of breast cancer patients and healthy controls quantified by flow cytometry and real-time PCR. This comparison showed a strong correlation between both methods.

In concordance to literature our comparison of controls and newly diagnosed breast cancer patients showed increased numbers of CEC in the cancer patients with both methods (Mancuso *et al*, 2001; Beerepoot *et al*, 2004). In real-time PCR the difference was more pronounced as in the flow cytometric detection. This could result from the difference in detection of dead cells with both methods: With flow cytometry also dead cells that still display intact morphology could be measured, but the mRNA in such cells is probably lost and therefore the real-time PCR will not detect these dead cells. This is supported by the previous finding that the amounts of VE-cadherin mRNA is strongly reduced in apoptotic endothelial cells compared to viable cells (Rabascio *et al*, 2004).

In the real-time approach we did not normalise to any housekeeping gene. For other real-time PCR analyses it is useful to normalise for housekeeping genes as *β*-actin or GAPDH to eliminate differences in cell numbers, RNA preparation and cDNA synthesis. However, for the quantification of CEC it is not useful to apply such a normalisation strategy. The normalisation to a housekeeping gene would result in the calculation of a ratio of CEC to leucocytes, since the housekeeping genes are expressed in all cells and the leucocytes are at least in a 1000-fold surplus compared to the CEC. Such a ratio would not reflect absolute CEC numbers in the blood samples, since the ratio also depends on the number of leucocytes which could be strongly influenced by infections or cancer therapies as chemotherapy or radiation. To get information about the real amounts of CEC in peripheral blood it would be necessary to take the leucocyte counts of the blood samples into account. To test the variations in our method, RNA was prepared several times individually from the same blood sample and with these separate samples real-time PCR analysis was performed. Since the variations between these samples were minimal (s.d. of *C*_t_-values: 0.28) a normalisation to a housekeeping gene and inclusion of the leucocyte numbers is not necessary in this analysis and would only result in increased inaccuracy instead of reduced variation. Nevertheless we recommend to include the measurement of a housekeeping gene as an internal control for individual RNA/cDNA quality.

The plasma levels of VEGF are often used as surrogate marker for angiogenesis. We therefore compared VEGF plasma concentrations with the amounts of CEC detected by flow cytometry and CD146 real-time PCR. We found a correlation between VEGF plasma levels and the CEC numbers. The comparison of VEGF levels in breast cancer patients and healthy controls did not show a significant difference as it was demonstrated for the CEC amounts. Also very high amounts of VEGF were found in two healthy controls. These observations could result from limitations of the ELISA measurement where factors as crossreacting antibodies or lipemic or haemolytic samples could falsify VEGF measurement. Thus, VEGF seems not to be an ideal surrogate marker for angiogenesis.

In conclusion, we could show that the quantification of CEC by real-time PCR for the endothelial cell marker CD146 is equivalent to four-colour flow cytometry analysis. Thus, CD146 real-time PCR is an easy and reliable alternative to quantify CEC in peripheral blood samples. This may considerably facilitate the determination of the angiogenic status of patients and the evaluation of antiangiogenic therapeutics.
